# SLC25A21 Suppresses Cell Growth in Bladder Cancer *via* an Oxidative Stress-Mediated Mechanism

**DOI:** 10.3389/fonc.2021.682710

**Published:** 2021-09-09

**Authors:** Yong Wang, Jiawen Gao, Shasha Hu, Weiting Zeng, Hongjun Yang, Hui Chen, Shuang Wang

**Affiliations:** ^1^Department of Urology, Jilin Province People's Hospital, Changchun, China; ^2^Department of Pathophysiology, College of Basic Medical Sciences, Jilin University, Changchun, China; ^3^Department of Pathology, Nanfang Hospital, Southern Medical University, Guangzhou, China; ^4^Department of Pathology, School of Basic Medical Sciences, Southern Medical University, Guangzhou, China

**Keywords:** SLC25A21, bladder cancer, α-ketoglutarate, ROS, apoptosis

## Abstract

**Background:**

Bladder cancer (BCa) is a commonly diagnosed malignancy worldwide that has poor survival depending on its intrinsic biologic aggressiveness and a peculiar radio- and chemoresistance features. Gaining a better understanding of tumorigenesis and developing new diagnosis and treatment strategies for BCa is important for improving BCa clinical outcome. SLC25 family member 21 (SLC25A21), a carrier transporting C5-C7 oxodicarboxylates, has been reported to contribute to oxoadipate acidemia. However, the potential role of SLC25A21 in cancer remains absolutely unknown.

**Methods:**

The expression levels of SLC25A21 in BCa and normal tissues were examined by real-time PCR and immunohistochemistry. Gain-of- and loss-of-function experiments were performed to detect the biological functions of SLC25A21 *in vitro* and *in vivo* by CCK-8 assay, plate colony formation assay, cell migration, invasion assay and experimental animal models. The subcellular distribution of substrate mediated by SLC25A21, mitochondrial membrane potential and ROS production were assessed to explore the potential mechanism of SLC25A21 in BCa.

**Results:**

We found that the expression of SLC25A21 was downregulated in BCa tissues compared to normal tissues. A significant positive correlation between decreased SLC25A21 expression and poor prognosis was observed in BCa patients. Overexpression of SLC25A21 significantly inhibited cell proliferation, migration and invasion and induced apoptosis *in vitro*. Moreover, the enhanced SLC25A21 expression significantly suppressed tumor growth in a xenograft mouse model. Furthermore, we revealed that SLC25A21 suppressed BCa growth by inducing the efflux of mitochondrial α-KG to the cytosol, decreasing to against oxidative stress, and activating the ROS-mediated mitochondrion-dependent apoptosis pathway.

**Conclusions:**

Our findings provide the first link between SLC25A21 expression and BCa and demonstrate that SLC25A21 acts as a crucial suppressor in BCa progression, which may help to provide new targets for BCa intervention.

## Background

Bladder cancer (BCa) is one of the most common malignancies of the urinary system, with approximately 900,000 new cancer cases and 250,000 mortalities worldwide each year (1). Approximately 70% of all newly diagnosed BCa cases are non-muscle-invasive BCa (nmiBCa). However, up to 30% of nmiBCa patients will relapse or develop progression to muscle-invasive BCa (miBCa) (2, 3). Additionally, in 5-15% of patients with BCa, unresectable or metastatic disease is found at the time of diagnosis (4). The poor survival of BCa patients is related to the intrinsic biologic aggressiveness of this tumor and its peculiar radio- and chemoresistance. Therefore, efforts to gain a deep understanding of the pathogenic mechanisms contributing to BCa carcinogenesis to identify new effective targets for therapy are strongly suggested.

Mitochondria are in fact the gatekeepers of the eukaryotic cell viability through regulating programmed cell death and controlling nuclear functions *via* the production of reactive oxygen species (ROS) (5), the modulation of calcium levels (6), and the trafficking of small molecule metabolites (7). The transfer of metabolites through mitochondrial membranes is a vital process that is highly controlled and regulated by the inner mitochondrial membrane (IMM). To fulfill its functions, a myriad of mitochondrial carriers are properly expressed, located and folded in the IMM (8). Mitochondrial carriers constitute a large family of transport proteins that play important roles in the intracellular translocation of metabolites, nucleotides and coenzymes. Solute carrier family 25 (SLC25) is a six-transmembrane-helix mitochondrial inner membrane protein family that includes 53 members (8). SLC25 facilitates the transport of molecules involved in the urea and citric acid cycle, oxidative phosphorylation, and iron metabolism among other processes (9). Growing evidence shows that some members of the SLC25 family can also be involved either directly or indirectly in pathological states, including cancer (10–13). The major role of SLC25 family member 21 (SLC25A21), also known as the oxoadipate carrier (ODC), is to mediate the import of 2-oxoadipate into the mitochondrial matrix in exchange for 2-oxoglutarate (α-ketoglutarate, α-KG) out. Previous studies have reported that the SLC25A21 defects may contribute to oxoadipate acidemia, a neurological disorder that is characterized by excessive amounts of oxoadipate and aminoadipate (14). Deletion of the 14q13.3 region, which contains the SLC25A21 gene, has also been reported in lung cancer (15). However, little is known about the potential role of SLC25A21 in cancer, especially BCa.

The present study was designed to explore the effects of SLC25A21 on BCa progression and the potential mechanisms. We identified that the expression levels of SLC25A21 were downregulated in BCa tissues by real-time PCR and immunohistochemistry. We further demonstrated the effects of SLC25A21 on cellular biological behaviors both *in vitro* and *in vivo* by gain- and loss-of-function experiments. We further presented evidence that SLC25A21 induces the efflux of mitochondrial α-KG to the cytosol and activates the ROS-mediated apoptosis pathway. Our findings provide novel insights into the roles of SLC25A21 in the tumorigenesis and progression of BCa, which may help to provide new therapeutic targets for BCa.

## Methods and Materials

### Ethics Statement

The use of tissues for this study has been approved by the ethics committee of Nanfang Hospital, Southern Medical University (Guangzhou, China). All of the patients signed an informed consent before the use of these clinical materials for research purposes. Moreover, the animal experiments were performed in strict accordance with the recommendations in the Guide for the Care and Use of Laboratory Animals of the National Institutes of Health. The protocol was approved by the Committee on the Ethics of Animal Experiments of Southern Medical University.

### Cell Lines and Tissue Specimens

The human normal ureter epithelial cell line (SV-HUC-1) and bladder cancer T24, EJ, BIU-87 and UMUC3 cells were obtained from American Type Culture Collection (ATCC, Manassas, VA, USA). UMUC3 cells were maintained in DMEM (Gibco, Gaithersburg, MD, USA) and T24, EJ and BIU-87 cells were maintained in RPMI-1640 medium (Gibco) supplemented with 10% fetal bovine serum (FBS Gibco) and 1% penicillin G sodium/streptomycin sulfate. SV-HUC-1 cells were cultured in F-12K medium (Gibco) with 10% FBS, 2 mmol/L L-glutamine and 1500 mg/L sodium bicarbonate. All of the cell lines were cultured at 37°C and 5% CO_2_ in a humidified incubator. All cell lines were used at early passages.

The tissue samples used for this study were obtained from randomly selected patients with a diagnosis of primary BCa from biopsy, transurethral resection of bladder tumor or radical cystectomy in Nanfang Hospital, Southern Medical University. Freshly frozen samples from BCa patients were selected for real-time PCR. 56 formalin-fixed paraffin-embedded BCa tissues and 20 adjacent non-tumor tissues were used to construct a tissue microarray (TMA) for investigating the expression of SLC25A21 protein using immunohistochemistry (IHC). One 0.6 mm tissues core had been punched out from representative regions of each tissue block, and was transferred into a TMA format as previously described (16). Tumor grade and stage assessments were made according to the World Health Organization (WHO) 2004 standard and Union for International Control Cancer (UICC) TNM system. The time of follow-up range from 3-82 months. The clinicopathological features of the case cohort are list in **Table 1**.

**Table 1:** Correlation between the clinicopathological features and expression of SLC25A21 in BCa.

Characteristics	n	SLC25A21 protein expression		
		low (%)	high (%)	*P* value
Gender				
Male	46	28 (60.1)	18 (39.9)	0.078
Female	10	9 (90)	1 (10)	
Age(years)				
<69	26	18 (69.2)	8 (30.8)	0.642
≥69	30	19 (63.3)	11 (36.7)	
Tumor diameter (cm)				
<5	34	22 (64.7)	12 (35.3)	0.788
≥5	22	15 (68.2)	7 (31.8)	
Tumor grade				
Low	18	9 (50.0)	9 (50.0)	0.080
High	38	28 (73.7)	10 (26.3)	
Invasion				
No	15	6 (40.0)	9 (60.0)	0.013
Yes	41	31 (75.6)	10 (24.4)	
T stage				
T_a_+T_is_+T_1_	17	7 (41.2)	10 (58.8)	0.008
T_2_	15	14 (93.3)	1 (6.7)	
T_3_+T_4_	24	16 (66.7)	8 (33.3)	
N stage				
N_0_	50	33 (66.0)	17 (34.0)	0.974
N_1~3_	6	4 (66.7)	2 (33.3)	

### GEO Data Analysis

Microarray datasets were downloaded from public GEO database (https://www.ncbi.nlm.nih.gov/geo/) and normalized using the robust multichip average (RMA) with R/Bioconductor packages including affy (17). GSE3167 and GSE68020 datasets are analyzed in present study. GSE3167 dataset includes superficial transitional cell carcinoma (sTCC) with surrounding CIS (13 patients), without surrounding CIS lesions (15 patients), in muscle invasive carcinomas (mTCC; 13 patients). GSE68020 includes 30 high-grade urothelial carcinoma and 20 benign tissues.

### RNA Isolation and Real- Time PCR

Total RNA from tissues and cell lines were extracted using Trizol reagent (Takara, Dalian, China) according to the manufacturer’s protocol. The cDNA was synthesized with 500 ng total RNA by using PrimeScript RT Reagent Kit (Takara). Real-time PCR was carried out using SYBR^®^ Rremix Ex TaqTM (Takara) as described previously (18). GAPDH was used as an internal control. The assay was performed in triplicate. All primer sequences are listed in **Supplement Information S1**.

### IHC Staining and Scoring

IHC was performed on 3-μm sections of paraffin-embedded tissue samples. The sections were deparaffinized with xylene and rehydrated with descending ethanol concentrations, and then were treated with Peroxidase Blocking Reagent (Dako, Glostrup, Denmark) for 5 min, followed by incubation with SLCA5A21 primary antibody (1:80, Affinity Biosciences, OH, USA, Cat# DF4172) at 4°C overnight. After incubation with the secondary antibody at room temperature for 1 h. Immunodetection was performed with the diaminobenzidine (DAB) reagent (Dako) according the manufacturer’s protocol, and the reaction time of each section was consistent, followed by counterstaining with hematoxylin.

The IHC-stained tissue sections were reviewed and scored separately in a double-blinded manner. Scores were determined based on both the intensity and proportion of SLC25A21-positive cells as described previously (19, 20). The staining score ranged from 0 to 4, corresponding to the percentage of immunoreactive tumor cells (0%, 1-25%, 26-50%, 51-75%, or 76-100%). The staining intensity scores were as follows: negative (0), weak (score = 1), medium (score = 2) or strong (score = 3). A total score of 0 to 12 was calculated by multiplying the staining degree score by the intensity score. The final staining score of ≥ 3 was considered to indicate high level of SLC25A21.

### Construction of Cell Lines With Stably Overexpressed SLC25A21

The full-length open reading frame of human SLC25A21 was amplified and cloned into the pcDNA3.1 vector. BCa cell lines EJ and T24 were transfected with pcDNA3.1-SLC25A21 using Lipofectamine 3000 reagent (Thermo Fisher Scientific, IL, USA). The empty pcDNA3.1 vector was used as the control. G418 was used to select stable SLC25A21-overexpressing cells. The expression levels of SLC25A21 were determined by real-time PCR and western blotting analysis.

### Oligonucleotide Transfection

The siRNAs targeting SLC25A21 and negative control siRNA (silencer negative control siRNA) were synthesized (GenePharma, Shanghai, China). Oligonucleotides transfection was performed with Lipofectamine 3000 following the manufacturer’s protocol. Target sequences for siRNAs were shown in **Supplement Information S2**.

### Cell Proliferation

Cell proliferation was assessed using the Cell Counting Kit-8 kit (Dojindo Laboratories, Kyushu Island, Japan) according to the manufacturer’s instructions. In brief, BCa cells were seeded in 96-well plates at 8 × 10^2^ cells per well. The results are reflected in the form of the absorbance optical density at 450 nm using a microplate reader (BioTek, VT, USA). The experiments were conducted in triplicate.

### Colony Formation Assay

Colony formation assays *in vitro* were performed according to standard protocols as previously described (21, 22). In brief, BCa cells were seeded in 6-well plates at 2 × 10^2^ cells per well and cultured in medium containing 10% FBS. After 2 weeks of culture, cell colonies were fixed with methanol for 30 min and stained with Giemsa for 15 min. The surviving colonies (≥50 cells per colony) were then counted under a microscope. The experiments were performed at least in triplicate.

### Cell Cycle Analysis and Apoptosis Analysis

Apoptosis analysis was performed by flow cytometry by a BD FACSCanto II Flow Cytometer (BD Bioscience, San Jose, CA, USA). The cells were collected and washed twice with ice-cold PBS, resuspended in 200 μl binding buffer and stained with the Annexin V-FITC Detection Kit (Genechem, Shanghai, China) according to the manufacturer’s instructions. Early apoptosis was determined based on the percentage of cells with annexin V^+^/PI^-^ staining, while late apoptosis was determine based on the percentage of cells with annexin V^+^/PI^+^ staining. The experiments were performed at least in triplicate.

For cell cycle, cells were harvested after 48 h transfection and fixed in ice-cold 70% ehanol at 4°C overnight. Cells were then resuspended in propidium iodide solution (Genechem) according to the manufacturer’s protocol and subjected to analysis using flow cytometry (BD).

### Wound Healing Assays

A wound healing assay was conducted to measure the migration of BCa cells. Cell migration was assessed by measuring the movement of cells into a scraped, acellular area by a 200 μL pipette tube in 6-well plates. The spread of wound closure was observed every 24 h. Migration ability was quantified by measuring the rate of cell migration toward the original wound field. The rate of cell migration were measured according to the formula: R_M_ (Rate of cell migration (μm/h)) = (W_i_ -W_f_)/t, W_i_ indicates initial wound width (μm), W_f_ indicates final wound width (μm) and t indicates duration of migration (hour), as described previously (23).

### Transwell Invasion Assays

Cell invasion assays were performed in Transwell chambers (BD Biosciences, San Jose, CA, USA) containing 8-μm pores in 24-well plates. A total of 1× 10^5^ cells were starved overnight and seeded into the upper chamber of the transwell, which had been pretreated with 60 μl Matrigel for 4 h, in 200 μl of serum-free medium. The lower chamber was supplemented with 500 μl of medium with 10% FBS. After incubation at 37°C for 24 to 72 h, noninvading cells in the upper chamber were scraped off with a cotton swab, and invading cells stuck to the lower surface of the membrane were fixed in 100% methanol for 30 min and stained with Giemsa solution for 15 min. The cells were counted randomly for five fields of each membrane under a light microscope. The experiments were performed at least in triplicate.

### *In Vivo* Functional Assays in Nude Mouse Models

Male, 4- to 6-week-old Balb/C-nu/nu nude mice were obtained from the Laboratory Animal Centre of Southern Medical University. Stably overexpressing SLC25A21 EJ cells and control cells were used to produce subcutaneous xenograft models. The nude mice were divided randomly into two groups (5 mice per group). A total of 2 × 10^6^ SLC25A21-overexpressing EJ cells or control cells were subcutaneously injected into the right flank of mice, respectively. Tumor growth was measured every 3 days with a digital caliper. Tumor volume was calculated according to the formula: V (volume) = (D × d^2^)/2 (D indicates the longest diameter and d indicates the shortest diameter). After 30 days of monitoring, the mice were sacrificed by cervical dislocation, and the xenografts were removed and subjected to histological examination. This investigation was carried out in strict accordance with ethical standards in the Guide for the Care and Use of Laboratory Animals of the National Institutes of Health. The protocol was approved by the Committee on the Ethics of Animal Experiments of Southern Medical University.

### Mitochondria Isolation and Determination of α-KG

Mitochondria extraction was conducted according to the instructions of the Mitochondria Isolation Kit (Beyotime Biotechnology, Jiangsu, China). Briefly, at least 2 × 10^7^ cells were harvested and then centrifuged at 600 × g at 4°C for 5 min. Mitochondrial isolation buffer was used to resuspend the pellet. The mixture was placed on ice for 15 min and then ground and centrifuged at 1000 × g at 4°C for 10 min. The supernatant was collected and centrifuged at 3500 × g at 4°C for 10 min. The pellet contained the mitochondria. The supernatant was collected and centrifuged at 12,000 × g at 4°C for 10 min. The supernatant contained the cytosolic fraction. Mitochondria were resuspended in mitochondria isolation buffer and centrifuged again at 3500 × g at 4°C for 10 min to obtain purified mitochondria. The levels of α-KG in the mitochondria and cytosolic fraction were determined using α-ketoglutarate (α-KG) enzyme-linked immunosorbent assay (ELISA) kits according to the documents of the manufacturer (JianglaiLab, Shanghai, China). In brief, 10 µL cell lysate and 40 µL sample diluent were added to the ELISA kit wells containing solid-phase antibody. After incubation at 37°C for 30 min, all the wells were washed five times with wash solution. Then, except for the blank wells, 50 µL horseradish peroxidase-conjugate reagent was added to each well. After incubation at 37°C for 30 min, all the wells were washed five times with wash solution again, and then 50 µL chromogen solution A and 50 µL chromogen solution B were added to each well. The reaction mixture was incubated at 37°C for 15 min in the dark. Finally, 50 µL stop solution was added to each well to stop the reaction, and read the absorbance at 450 nm within 15 min. The concentrations of α-KG in the mitochondria and cytosolic fraction were calculated according to the standard curve prepared following the direction of the kit. Data were expressed as mean ± standard deviation (SD) of three separate measurements.

### Mitochondrial Membrane Potential (*Δψm*)

Changes in the Δψm during the early stages of apoptosis were assayed using the Mitochondrial Membrane Potential Assay kit with JC-1 (Beyotime, Shanghai, China) as described previously (24). Briefly, 5 × 10^4^ cells were harvested and incubated with JC-1 at 37°C for 20 min in the dark. The stained cells were washed with ice-cold working solution twice and then analyzed by flow cytometry (BD). JC-1 aggregates in the polarized mitochondrial matrix and forms J-aggregates, which emit red fluorescence at 595 nm when excited at 525 nm. However, JC-1 cannot aggregate in the depolarized mitochondrial matrix and exists as JC-1 monomers, which emit green fluorescence at 525 nm when excited at 485 nm. Mitochondrial depolarization is indicated by a decrease in the red/green fluorescence intensity ratio.

### ROS Production

The ROS levels were detected with the Human ROS ELISA Kit and analyzed with a microplate reader according to the documents of the manufacturer (JianglaiLab, Shanghai, China).

### Western Blotting

Cells were lysed in prechilled RIPA buffer containing phosphatase inhibitors, protease inhibitors and PMSF. The protein extracts were loaded on each line of a 10% SDS-PAGE gel and transferred to polyvinylidene fluoride (PVDF) membranes (Millipore, Darmstadt, Germany). The membranes were blocked in 5% skimmed milk in 1 × PBS-T (0.5% Tween-20) and incubated overnight at 4°C with the following primary antibodies: anti-SLC25A21 (1:500; Affinity Biosciences), anti-cytochrome C (1:500; Affinity Biosciences), anti-caspase-9 (1:800; Affinity Biosciences), anti-cleaved caspase-9 (1:800; Affinity Biosciences), anti-caspase-3 (1:800; Affinity Biosciences), and anti-cleaved caspase-3 (1:800; Affinity Biosciences). Anti-tubulin (1:1000; Proteintech Group, Wuhan, China) was used as protein-loading control. Blots were incubated with HRP-conjugated secondary antibodies for 1 h at room temperature, and visualized with ECL Western Blotting Substrate (ThermoFisher Scientific).

### Statistical Analysis

All statistical analyses were performed using SPSS version 16.0 software (SPSS, Chicago, Illinois, USA). Differences between groups were identified using a two-tailed Student’s *t*-test. Associations between SLC25A21 expression and clinicopathological characteristics were determined by the *χ* ^2^ test. Survival curves were plotted by the Kaplan–Meier method and compared by the log-rank test. The significance of various variables for survival was analyzed by the Cox proportional hazards model for multivariate analyses. A probability value of 0.05 or less was considered to be significant.

## Results

### SLC25A21 Is Downregulated in BCa and Is Negatively Associated With Poor Prognosis in BCa Patients

**Figure 1:**
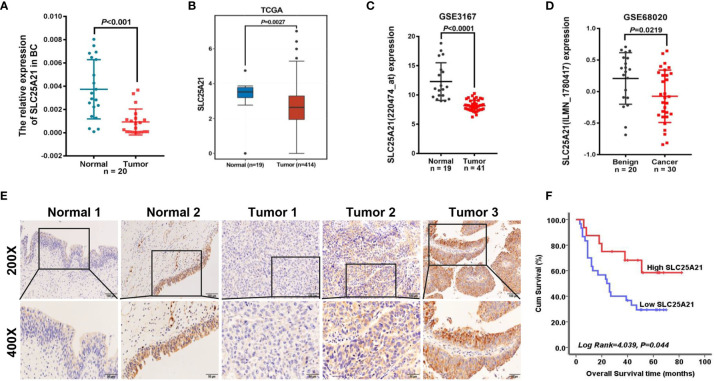
SLC25A21 is downregulated in BCa and negatively associated with poor prognosis in BCa patients. **(A)** Expression levels of SLC25A21 mRNA in BCa and pair-matched noncancerous tissue samples. **(B–D)** Analysis of SLC25A21 expression in BCa compared with noncancerous tissue according to RNA-seq in TCGA database and BCa microarray profile (GSE3167 and GSE68020) **(E)** Expression analysis of SLC25A21 protein in BCa and normal bladder mucosa tissues by IHC. Scale bars, 100 μm (200x) or 50 μm (400x). **(F)** Correlation between SLC25A21 protein expression and overall survival in BCa patients (log-rank *P* =0.044).

To evaluate the expression levels of endogenous SLC25A21 in BCa, we first examined SLC25A21 mRNA expression in 20 BCa tissues and their pair-matched noncancerous tissue samples using real-time PCR. As shown in **Figure 1A**, SLC25A21 mRNA was significantly downregulated in BCa tissues compared to nontumor tissues (*P*<0.001). Meanwhile, SLC25A21 mRNA levels was down-regulated in all the BCa cell lines compared with a normal cell line (SV-HUC-1) cells (*P <*0.05, **Supplementary Figure S1**). To further confirm the levels of SLC25A21 expression in BCa, we also analyzed the SLC25A21 expression in public datasets from The Cancer Genome Atlas (TCGA) and the Gene Expression Omnibus (GEO) database. By analyzing the TCGA, we also found that SLC25A21 was lower expressed in transcript levels in BCa than in normal bladder mucosa according to a Gene Expression Profiling Interactive Analysis website (GEPIA) (gepia.cancer-pku.cn/) (**Figure 1B**). In addition, consistent with our results, SLC25A21 was expressed at lower levels in BCa tissues than in normal tissues in GSE3167 and GSE68020 datasets (**Figures 1C, D**).

To evaluate whether SLC25A21 expression was associated with the clinicopathologic features of BCa patients, we further performed immunostaining with SLC25A21 antibody in tissue microarrays using IHC and observed that SLC25A21 was highly expressed in 60.00% (12 of 20) of normal tissues and only in 33.93% (19 of 56) of BCa samples. The levels of SLC25A21 were significantly downregulated in BCa tissues (*P* =0.042, **Figure 1E**). The SLC25A21 immunoreactivity scores showed that the lower levels of SLC25A21 expression were associated with tumor invasion and T stage (**Table 1**). In addition, we also analyzed the prognostic significance of SLC25A21 expression in patients with BCa. As shown in **Figure 1F**, it was revealed that low expression of SLC25A21 was significantly correlated with poor survival in BCa patients (log rank *P* = 0.044). Taken together, these findings suggest that SLC25A21 expression is downregulated in BCa and that low SLC25A21 expression is a poor prognostic biomarker for patients with BCa, which indicates that SLC25A21 might behave as a suppressor in BCa.

### Overexpression of SLC25A21 Inhibits the Growth, Migration and Invasion of BCa Cells *In Vitro*

**Figure 2:**
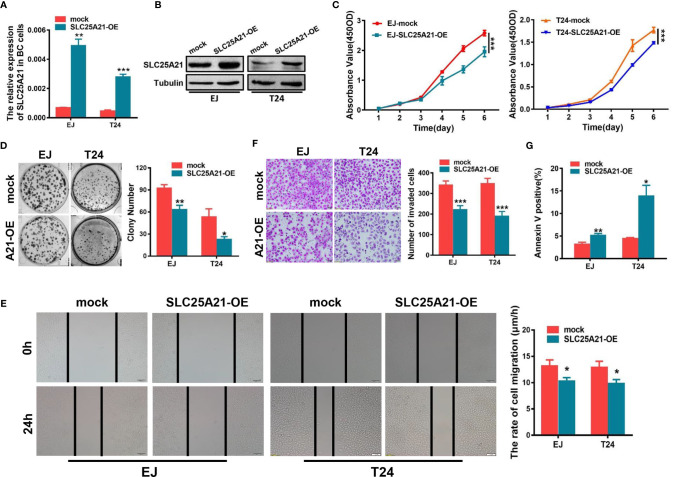
SLC25A21 overexpression inhibits the cell proliferation, migration and invasion and induces the apoptosis of BCa cells *in vitro*. **(A, B)** Increased expression of SLC25A21 after transfection of the pcDNA3.1-SLC25A21 vector was confirmed in two BCa cell lines by real-time RT-PCR **(A)** and western blotting **(B)**. **(C)** SLC25A21 overexpression inhibits the proliferation of EJ and T24 cells. **(D)** SLC25A21 overexpression decreased the colony formation capacity of EJ and T24 cells. **(E)** The migration ability of BCa cells overexpressing SLC25A21 was decreased, as indicated by the wound healing assay. **(F)** Matrigel invasion chamber assays showed that SLC25A21 overexpression inhibited the invasiveness of both EJ and T24 cells. Representative images (left) and quantitative analyses (right) are shown. **(G)** SLC25A21 overexpression induced the apoptosis rate relative to control cells. Data are presented as the mean ± SD. The results were reproducible in three independent experiments. *P < 0.05, **P < 0.01, and ***P < 0.001.

To investigate whether SLC25A21 plays an important role in the biological behavior of BCa carcinogenesis, we established stable SLC25A21-overexpressing BCa cells and SLC25A21-depleted BCa cells. EJ and T24 cells which have relatively low endogenous mRNA expression were used to establish two BCa cell lines with stable SLC25A21 overexpression. Real-time PCR and western blotting analyses revealed that the relative level of SLC25A21 in BCa cells was significantly increased compared to that in control cell lines (**Figures 2A, B**). CCK8 assays revealed a significantly slower proliferation rate in SLC25A21-overexpressing EJ and T24 cells than in control cells (*P*<0.001, **Figure 2C**). In the colony formation assay, both SLC25A21 overexpression obviously suppressed colony formation in both BCa cell lines (EJ, *P* = 0.0028 and T24, *P* = 0.0103, **Figure 2D**).

We also examined the effects of SLC25A21 overexpression on BCa cell migration and invasion. Wound healing assays were performed to explore the effects on the migration ability of BCa cells after enforcing the expression of SLC25A21. The results suggested that SLC25A21 upregulation inhibited the rate of migration of BCa cells (EJ cells, *P*=0.0211 and T24 cells, *P*=0.0181, **Figure 2E**). Cell invasion analysis demonstrated that the exogenous SLC25A21 overexpression in two different BCa cell lines significantly inhibited cancer cell invasion through Matrigel, a basement-membrane- like extracellular matrix (*P*<0.001, **Figure 2F**).

### Overexpression of SLC25A21 Induces Cell Apoptosis in BCa Cells

Based on the results of CCK-8 and colony formation assays, we next investigated the potential mechanisms underlying the growth-inhibitory effects of SLC25A21 overexpression. The results of the flow cytometry cell cycle assays in two SLC25A21-overexpressing cell lines were inconsistent. SLC25A21 overexpression induced G_1_ phase cell cycle arrest in EJ cells (*P*=0.0043) and reduced the G_1_ phase cell population in (*P*=0.0079, **Supplementary Figure S2**). However, the upregulation of SLC25A21 expression resulted in an increased percentage of early apoptosis (EJ, *P* = 0.0092 and T24, *P* = 0.0138, **Figure 2G** and **Supplementary Figure S3**) in both BCa cell lines compared with controls. In short, these data indicate that the SLC25A21-mediated decrease in cell proliferation is modulated by apoptosis, rather than by the G_1_-S checkpoint.

### SLC25A21 Overexpression Suppressed Tumor Growth of BCa Cells *In Vivo*

**Figure 3:**
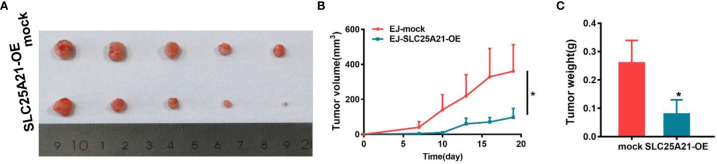
SLC25A21 overexpression suppresses BCa cell proliferation *in vivo*. **(A)** SLC25A21 overexpression inhibited subcutaneous tumor formation in nude mice. EJ cells with SLC25A21 upregulation and control cells were inoculated into nude mice (n = 5 per group). **(A)** The xenografts of 30 days after ectopic-subcutaneous implantation of with SLC25A21 overexpressing and control cells into nude mice. **(B, C)** The effect of SLC25A21 overexpression on BCa tumor growth was evaluated based on tumor volume **(B)** and tumor weight **(C)** in the two groups. *P < 0.05.

In light of our *in vitro* findings, we also assessed the effects of SLC25A21 overexpression on the growth of xenograft tumors *in vivo*. EJ cells with SLC25A21-overexpressing and control cells were subcutaneously inoculated into nude mice. As shown in **Figures 3A, B**, compared with control cells, SLC25A21 overexpression significantly inhibited BCa cell growth *in vivo* (*P=0.0392*). The tumor weight was lower in the stable SLC25A21overexpression group than in the control groups (*P=0.0293*, **Figure 3C**). These results suggest that SLC25A21 exerts a significant inhibitory effect on BCa tumorigenesis *in vivo*.

### SLC25A21 Knockdown Promotes BCa Cell Proliferation, Migration and Invasion and Inhibits Cell Apoptosis *In Vitro*

**Figure 4:**
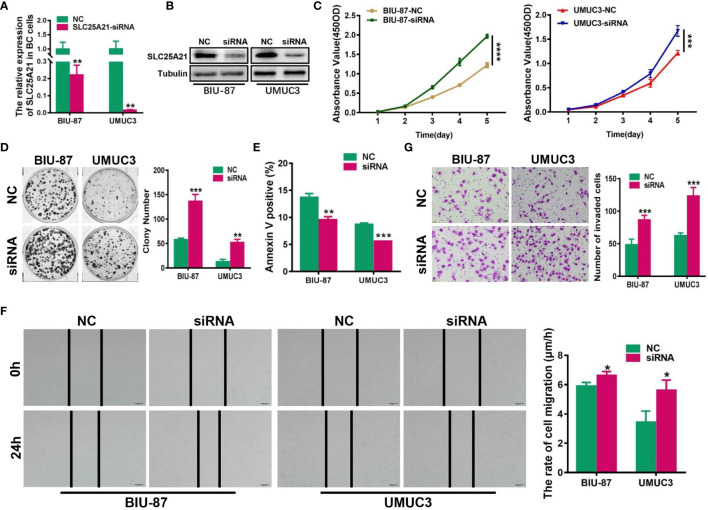
siRNA-mediated knockdown of SLC25A21 promotes BCa cell proliferation, migration and invasion, and represses cell apoptosis *in vitro*. **(A, B)** Depletion of SLC25A21 after transient transfection of siRNA targeting SLC25A21 was confirmed in two BCa cell lines by real-time RT-PCR **(A)** and western blotting **(B)**. **(C, D)** The effects of SLC25A21 downregulation on BCa cell proliferation by CCK-8 **(C)** and colony formation assays **(D)**. **(E)** The effects of SLC25A21 depletion on cell apoptosis **(E)** by flow cytometry. **(F)** The effects of SLC25A21 depletion on the migration ability of BCa cells by using a wound healing assay. **(G)** Invasion assays were used to determine the effects of SLC25A21 depletion on the invasion ability of BCa cells. Representative images (left) and quantitative analyses (right) are shown. Data are expressed as the means ± SD of three independent experiments. *P < 0.05, **P < 0.01, ***P < 0.001, and ****P < 0.0001.

To assess the effect of reducing SLC25A21 expression in BCa, we next knocked down SLC25A21 levels by siRNA transfection in BIU-87 and UMUC3 cells, which have relatively high endogenous SLC25A21 expression. A considerable reduction in SLC25A21 was observed in both SLC25A21-depleted BCa cell lines (**Figures 4A, B**). The results of CCK8 and colony formation assays showed that both BCa cell lines with SLC25A21 knocked down had a significant growth advantage over the respective cells transfected with negative control siRNA (*P*<0.01, **Figures 4C, D**). Moreover, SLC25A21 knockdown inhibited cell apoptosis in BIU-87 (*P*=0.0014) and in UMUC3 cells (*P*<0.0001, **Figure 4E**). In addition, markedly higher migration rates and stronger invasiveness were observed in SLC25A21-depleted BIU-87 and SLC25A21-depleted UMUC3 cells, as revealed by the wound healing assay (BIU-87 cells, *P*=0.0254 and UMUC3 cells, *P*=0.0214, **Figure 4F**) and Transwell migration assay (*P*<0.0001, **Figure 4G**).

### SLC25A21 Overexpression Induces Cell Apoptosis by the α-KG-Mediated ROS Pathway in BCa

**Figure 5:**
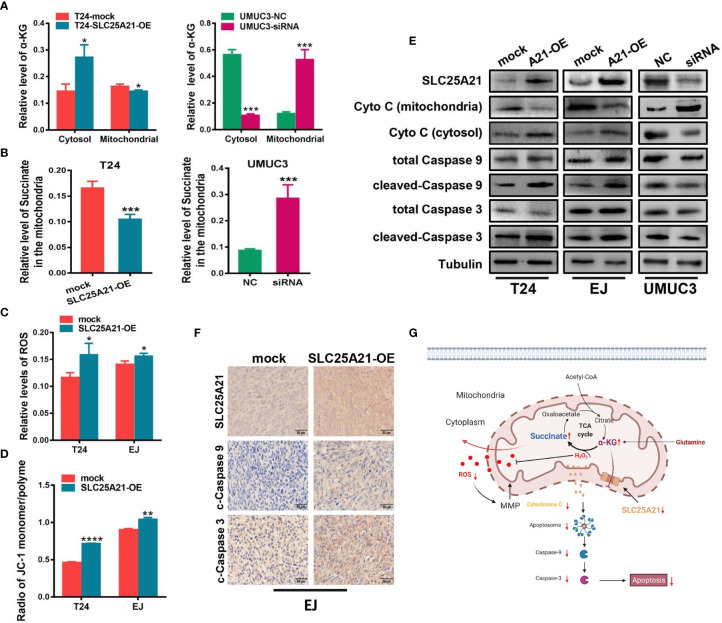
SLC25A21 overexpression induces cell apoptosis by the α-KG-mediated ROS pathway in BCa. **(A)** SLC25A21 promoted the efflux of α-KG from the mitochondria to the cytosol. **(B)** The levels of α-KG in the mitochondria affected succinate production. **(C)** The upregulation of SLC25A21 promoted ROS accumulation in BCa cells. **(D)** The upregulation of SLC25A21 decreased Δψm in both BCa cell lines. **(E)** Western blot assays showed that SLC25A21 induced cyto C transfer from the mitochondria to the cytosol and increased the activation of caspase-9 and caspase-3 in BCa cells. **(F)** Immunohistochemistry showed that SLC25A21 increased the activation of caspase-9 and caspase-3 in BCa xenograft tissues. **(G)** Schematic diagram showing the mechanism of action of SLC25A21 on cell apoptosis in BCa. The results were reproducible in three independent experiments. *P < 0.05, **P < 0.01, ***P < 0.001 and ****P < 0.0001.

We next studied the molecular mechanisms by which SLC25A21 affects proliferation. Because the function of SLC25A21 is linked to the transport of C5-C7 oxodicarboxylates, including α-KG, across the IMM, we speculated that aberrant SLC25A21 expression directly changes α-KG levels in the mitochondria. Therefore, we assessed the subcellular distribution of α-KG mediated by SLC25A21. SLC25A21 overexpression significantly increased α-KG levels in the cytosol and decreased α-KG levels in the mitochondria in T24 cells (cytosol, *P*=0.0150 and mitochondria, *P*=0.0284, **Figure 5A**, left). Moreover, SLC25A21 knockdown mediated by siRNA significantly elevated α-KG levels in the mitochondria of UMUC3 cells compared those after transfection with a negative control siRNA (*P*<0.001, **Figure 5A**, right). The oxidative metabolism of α-KG produces succinate in the mitochondria. Indeed, decreased levels of the succinate were observed in the mitochondria of BCa cells with SLC25A21 overexpressing (*P*=0.0032, **Figure 5B**, left). In contrast, the levels of succinate in the mitochondria were reduced in SLC25A21-depleted UMUC3 cells, further supporting the view that upregulation of SLC25A21 promoted the efflux of α-KG from mitochondria (*P*=0.0028, **Figure 5B**, right).

Because ROS generation is augmented by mitochondrial dysfunction, we next investigated whether SLC25A21 induces ROS production. The results showed that with the reduction of α-KG levels in the mitochondria, the upregulation of SLC25A21 in the two cell lines resulted in significantly higher ROS accumulation compared with the corresponding control group, which indicated a weaker ability to survive oxidative stress (T24 cells, *P*=0.0341 and EJ cells, *P*=0.0348, **Figure 5C**). We also evaluated the effects of SLC25A21 on Δψm in both SLC25A21-overexpressing cells by flow cytometry. As shown in **Figure 5D**, a decreased Δψm was observed in both SLC25A21-overexpressing BCa cell lines. T24 cells with SLC25A21-overexpressing exhibited a Δψm decline of more than 36.3% (*P*<0.0001), and EJ cells showed a 13.3% decline (*P*=0.0012) compared to the control. It has been reported to participate in triggering caspase-dependent apoptosis by regulating Δψm. The results of western blotting showed that SLC25A21 induced cytochrome C (cyto C) transfer from mitochondria to the cytosol in BCa cells. In addition, we also found that SLC25A21 overexpression increased the activation of caspase-3 and caspase-9 in BCa cell lines and xenografts tissues (**Figures 5E, F**), suggesting that SLC25A21 triggers mitochondrial apoptotic pathways in BCa. Hence, these data indicate that SLC25A21 activates the mitochondrial apoptosis pathway by inducing α-KG efflux from the mitochondria to the cytosol, and then increasing ROS levels, thereby activating cell apoptosis (**Figure 5G**).

## Discussion

In the present study, we illustrated that the downregulation of SLC25A21 plays a key role in promoting human BCa development. Our data showed that SLC25A21 is strongly downregulated in BCa tissues and is especially positively correlated with an invasion and poor outcome in BCa patients. We validated SLC25A21 as an important tumor suppressor gene in BCa by gain- and loss-of-function experiments *in vitro* and *in vivo*. Interestingly, we also found that SLC25A21 promoted the efflux of α-KG from the mitochondria and increased ROS levels, resulting in the activation of the mitochondrial apoptosis pathway. Thus, our study provides strong evidence supporting the tumor suppressor roles of SLC25A21 in BCa.

SLC25 is a large family of nuclear-encoded transporters embedded in the IMM and, in a few cases, other organelle membranes. The members of this superfamily are widespread in eukaryotes and are involved in numerous metabolic pathways and cell functions (9). In recent years mutations in the SLC25 genes have been shown to be responsible for some diseases (9), highlighting the important role of SLC25 in physiological and pathological implications.

Human SLCA25A21 is ubiquitously expressed with very little variation between tissues (14). The best substrates for SLC25A21 are 2-oxoadipate and α-KG (14). The physiological role of SLC25A21 involves in 2-oxoadipate acidemia, which is accompanied by the accumulation and excretion of large amounts of 2-oxoadipate, 2-aminoadipate and 2-hydroxyadipate in urine (14). Harris and colleagues first reported that the deletion of SLC25A21 appears to be associated with lung cancer (15). However, the effects and mechanisms responsible for these diseases have not been characterized. In the present study, we revealed that the SLC25A21 expression are significantly downregulated in BCa tissues compared with normal bladder epithelial tissues, in accordance with the results from our real-time RT-PCR, IHC and TCGA and GSEA database analyses, and we determined that low expression of SCL25A21 is significantly associated with invasion and poor survival in BCa patients. These data indicate that the downregulation of SLCA25A21 expression could serve as a predictor of a higher risk of BCa progression.

To highlight the function of SLC25A21 in BCa, we further explored the critical effects of SLC25A21 on cell behaviors in BCa by gain- and loss-of-function experiments. Our results identified that SLC25A21 acts as a tumor suppressor results from the overexpression of SLC25A21 inhibits cell growth and facilitates cell apoptosis in BCa *in vitro* and *in vivo*. To the best of our knowledge, our study provides the first evidence that SLC25A21 plays a critical role in tumors, especially BCa, which suggesting SLC25A21 as a potential predictor and therapeutic target for BCa progression.

Many new functions have been ascribed to SLC25 in tumors from data generated over the last year (10, 12). For example, SLC25A22, a glutamate carrier that facilitates the transport of glutamate across the IMM into the mitochondria matrix, promotes cell proliferation and tumor progression of colorectal cancer with KRAS mutations *via* intracellular synthesis of aspartate (10). The knockdown of SLC25A10 changes the growth properties to a less malignant phenotype and causes increased glutamine dependency and sensitivity to oxidative stress in human lung adenocarcinoma cells, suggesting SLC25A10 as a novel target for anticancer strategies (13). However, the mechanisms by which SLC25A21 mediates the progression of BCa remain unknown.

The main physiological function of human SLC25A21 is probably to catalyze the uptake of 2-oxoadipate into the mitochondria and the efflux of α-KG from the mitochondria to the cytosol. α-KG is an intermediate of the tricarboxylic acid cycle (TAC) that plays important roles in cell metabolism and physiology. As a biological substance, α-KG is essential for the oxidation of fatty acids, amino acids and glucose, and serves as the carbon skeleton for the synthesis of glutamate and glutamine (25). Additionally, it has been established that α-KG can act as a true antioxidant, because it directly reacts with hydrogen peroxide (H_2_O_2_) to form succinate, water, and carbon dioxide (26, 27). The supplementation of α-KG can directly or indirectly stimulate endogenous antioxidant defense (28, 29). Therefore, we speculated that SLC25A21 depletion-mediated α-KG arrest in mitochondria may reduce ROS generation in BCa. Indeed, when higher levels of mitochondrial α-KG are present in SLC25A21-depleted BCa cells, ROS production is significantly decreased, accompanied by an increase in succinate in the mitochondria and *vice versa.* These results suggest that SLC25A21 can directly protect against oxidative stress by restricting the efflux of α-KG in the mitochondria.

ROS may exhibit a dual role in promoting or suppressing cancer formation. Although upregulation of ROS is known to promote tumorigenesis, growing evidence suggests that excessive accumulation of ROS leads to cell apoptosis (30, 31). Apoptosis, a type of programmed cell death, is an important pathway for regulating homeostasis and morphogenesis and is associated with various diseases, especially cancer. Apoptosis is controlled by two principal pathways, including the death receptor-mediated extrinsic pathway and the mitochondrion-dependent intrinsic pathway (30). The intrinsic pathway is much more protean but mitochondrial membrane permeabilization (MMP) is a crucial step in the signaling cascade. MMP is triggered by a variety of stimuli, such as hypoxia, oxidative stress, DNA damage, and so on. All of them can induce Δψm loss in the mitochondria. The mitochondrial apoptosis pathway starts with mitochondrial depolarization, triggering the release of cyto C into the cytosol, thereby activating caspase-9 and caspase-3, and ultimately leading to apoptosis (32). Here, we also found that SLC25A21 reduced MMP and induced the subsequent following efflux of mitochondrial cyto C to the cytosol and activation of caspase 9 and caspase 3. The elevation of intracellular ROS levels mediated SLC25A21 overexpression-induced apoptosis in BCa cell lines, which may explain why SLC25A21 overexpression decreased the cell proliferation and colony formation ability of BCa cells *in vitro* and *in vivo*. In addition, our results also revealed that SCL25A21 downregulation is significantly associated with invasion state of BCa patients and promoted tumor cell migration and invasion. Recently, Wu et al. founded that cancer-derived succinate promotes macrophage polarization and cancer metastasis in murine and human lung cancer cells (33). Moreover, previous studies have reported that the accumulation of fumarate in mouse and human cells elicits an epithelial-to-mesenchymal-transition (EMT) (34). Frezza et al. reported that the fumarate inhibits Tet-mediated demethylation of a regulatory region of the antimetastastic miRNA cluster, leading to the expression of EMT-related transcription factors and enhanced migratory properties in renal cancer (35). In TAC reaction, α-KG is oxidized and decarboxylated to succinyl CoA mediated by the α-KG dehydrogenase complex, which in turn producing succinate. Then, succinate is oxidized and hydrated to fumarate. Here, we observed that higher levels of mitochondrial α-KG promoted the accumulation of succinate in the mitochondria in SLC25A21-depleted BCa cells. Therefore, we speculated that SLC25A21 depletion-mediated α-KG arrest in mitochondria may result in an increase of succinate and fumarate, thereby promoting cell invasion, and metastasis in BCa. These data indicate that the downregulation of SLCA25A21 expression could serve as a predictor of a higher risk of BCa progeression. The mechanisms by which SLC25A21 mediates invasion inhibition present an interesting issue for further investigation.

## Conclusions

Our research demonstrated that SLC25A21 is downregulated in BCa tissues and that its downregulation might play a promoting role in BCa tumorigenesis and progression through the ROS-mediated mitochondrion-dependent apoptosis pathway. These results indicate that the overexpression of SLC25A21, which specifically induces ROS-mediated apoptosis in cancer cells, could be used to develop a highly effective treatment strategy for BCa patients.

## Data Availability Statement

The original contributions presented in the study are included in the article/**Supplementary Material**. Further inquiries can be directed to the corresponding author.

## Ethics Statement

The use of tissues for this study has been approved by the ethics committee of Nanfang Hospital, Southern Medical University by NFEC-2021-108. The animal study was reviewed and approved by SCXK2016-0041.7.

## Author Contributions

SW designed research. YW, JG, SH, WZ, and HC contributed in the experimental procedures. HY and SW analyzed data. SW and HY supervised all the work. All authors contributed to the article and approved the submitted version.

## Funding

This work was supported by the National Natural Science Foundation of China (81972754), the Guangdong Basic and Applied Basic Research Foundation (2019A1515012226) and Guangdong Province Rural Science and Technology special commissioner project (KTP20190277).

## Conflict of Interest

The authors declare that the research was conducted in the absence of any commercial or financial relationships that could be construed as a potential conflict of interest.

## Publisher’s Note

All claims expressed in this article are solely those of the authors and do not necessarily represent those of their affiliated organizations, or those of the publisher, the editors and the reviewers. Any product that may be evaluated in this article, or claim that may be made by its manufacturer, is not guaranteed or endorsed by the publisher.

## Abbreviations

BCa, Bladder cancer; nmiBCa, non-muscle-invasive BCa; miBCa, muscle-invasive BCa; ROS, reactive oxygen species; IMM, the inner mitochondrial membrane; SLC25, Solute carrier family 25; SLC25A21, SLC25 family member 21; α-KG, α-ketoglutarate; *Δψm*, Mitochondrial membrane potential; MMP, mitochondrial membrane permeabilization; TCGA, The Cancer Genome Atlas; GEO, the Gene Expression Omnibus.
