# Supplementary material for: SLC25A21 Suppresses Cell Growth in Bladder Cancer via an Oxidative Stress-Mediated Mechanism
Source: Front Oncol. 2021 Sep 9;11:682710. doi: 10.3389/fonc.2021.682710 (PMC8458862; doi:10.3389/fonc.2021.682710)
Supplement: Supplementary file 1 [file DataSheet_1.docx]

Supplement Information

S1. PCR Primer

| SLC25A21: |  |
| --- | --- |
| Forward primer: | 5'- ATTTGGGATTGGTCTTCT-3' |
| Reverse primer: | 5'- GACTGTTGCCATTGTTTT-3' |
| GAPDH: |  |
| Forward primer: | 5'- ACAGTCAGCCGCATCTTCTT-3' |
| Reverse primer: | 5'- GACAAGCTTCCCGTTCTCAG-3' |

S2. siRNA sequence

| SLC25A21 siRNA: | 5'-GGAGA GAUCA AGUAC AGAATT-3' |
| --- | --- |
| NC siRNA: | 5’-ACGUGACACGUUCGGAGAATT-3’ |

**Figure S1**.


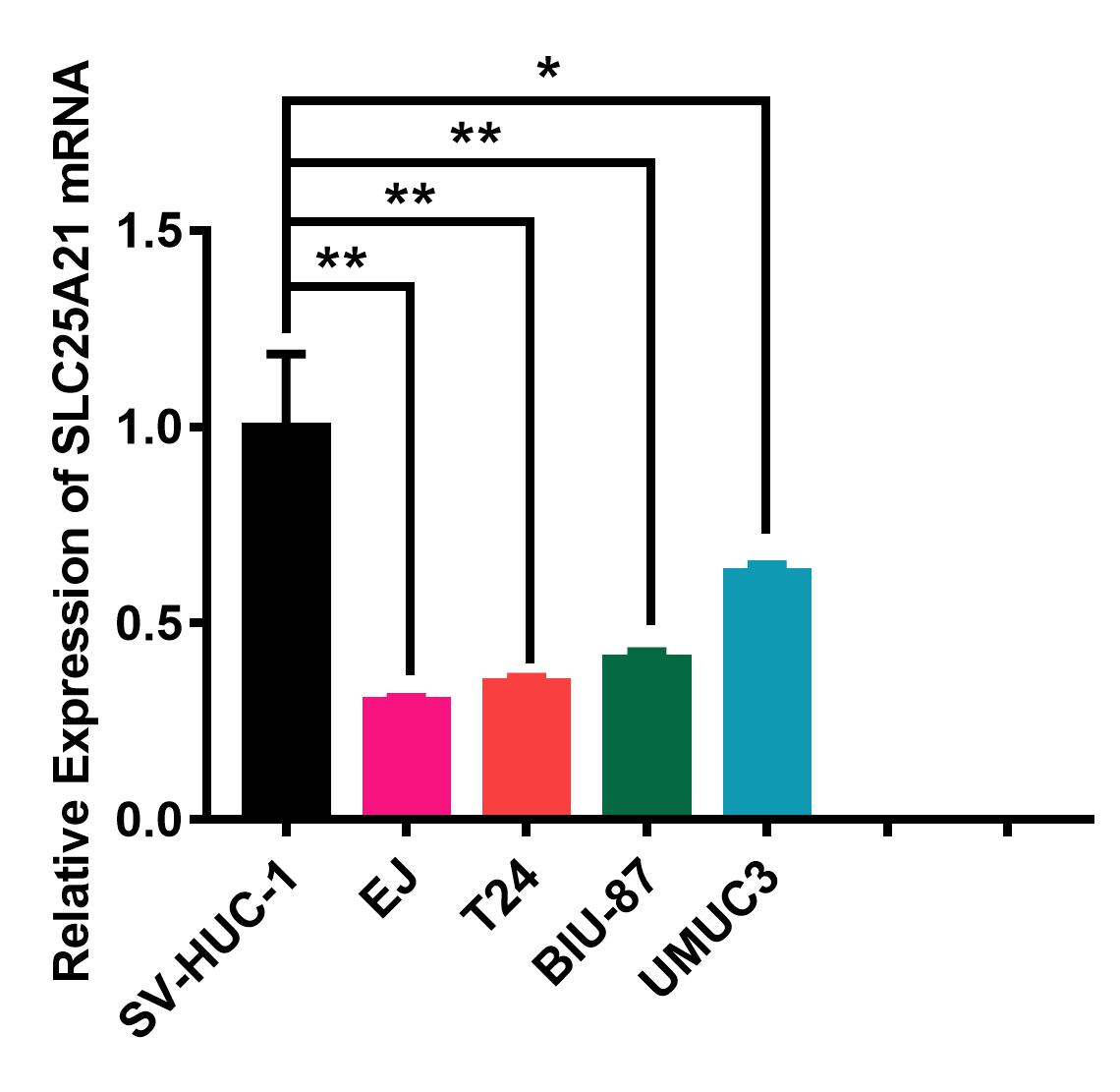


**Figure S1.** **The expression levels of SLC25A21 mRNA in BCa and normal cell lines.**

**Figure S2**.


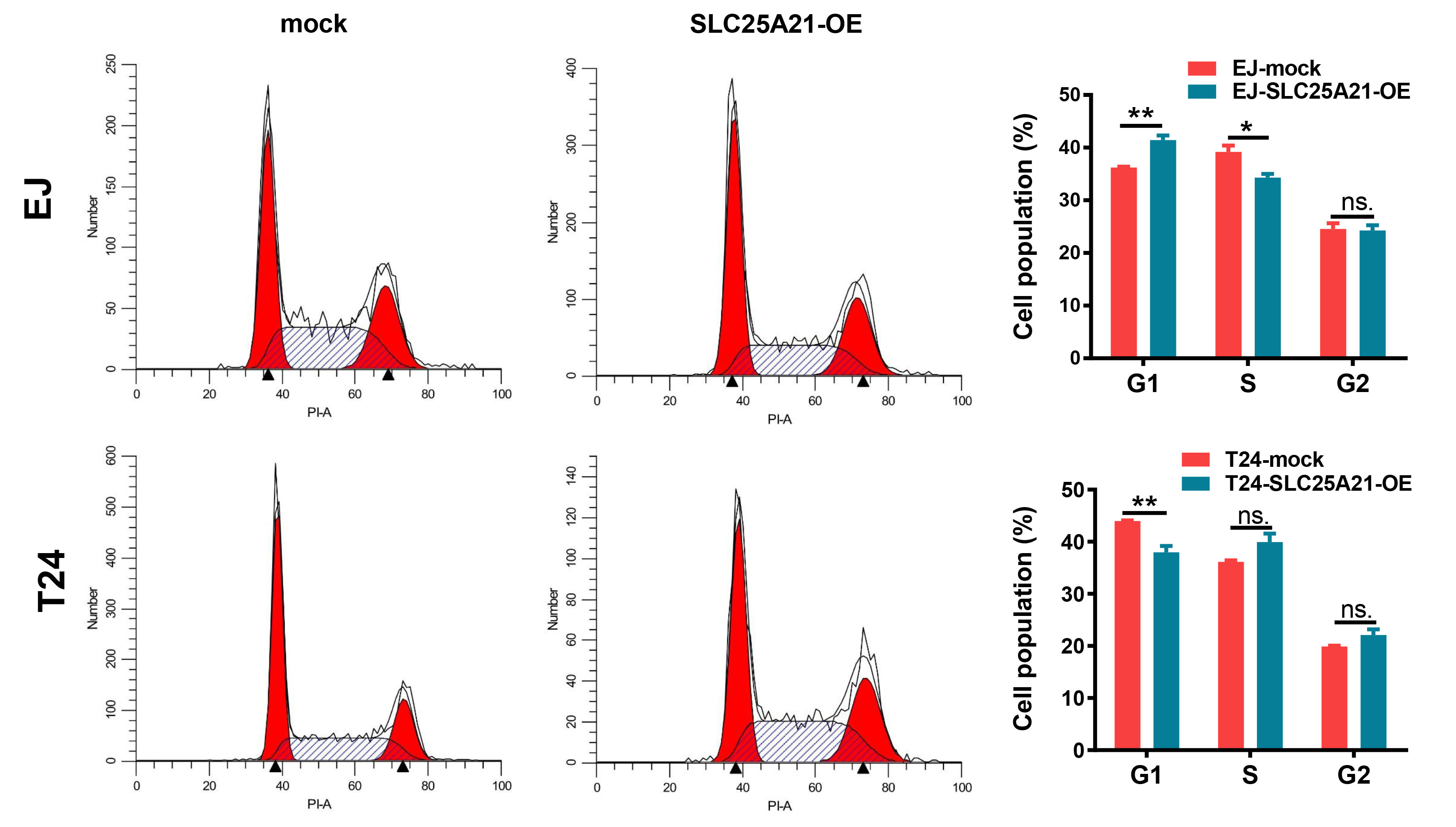


**Figure S2.** **The effect of SLC25A21 overexpression on cell cycle in BCa cells**

**Figure S3**.


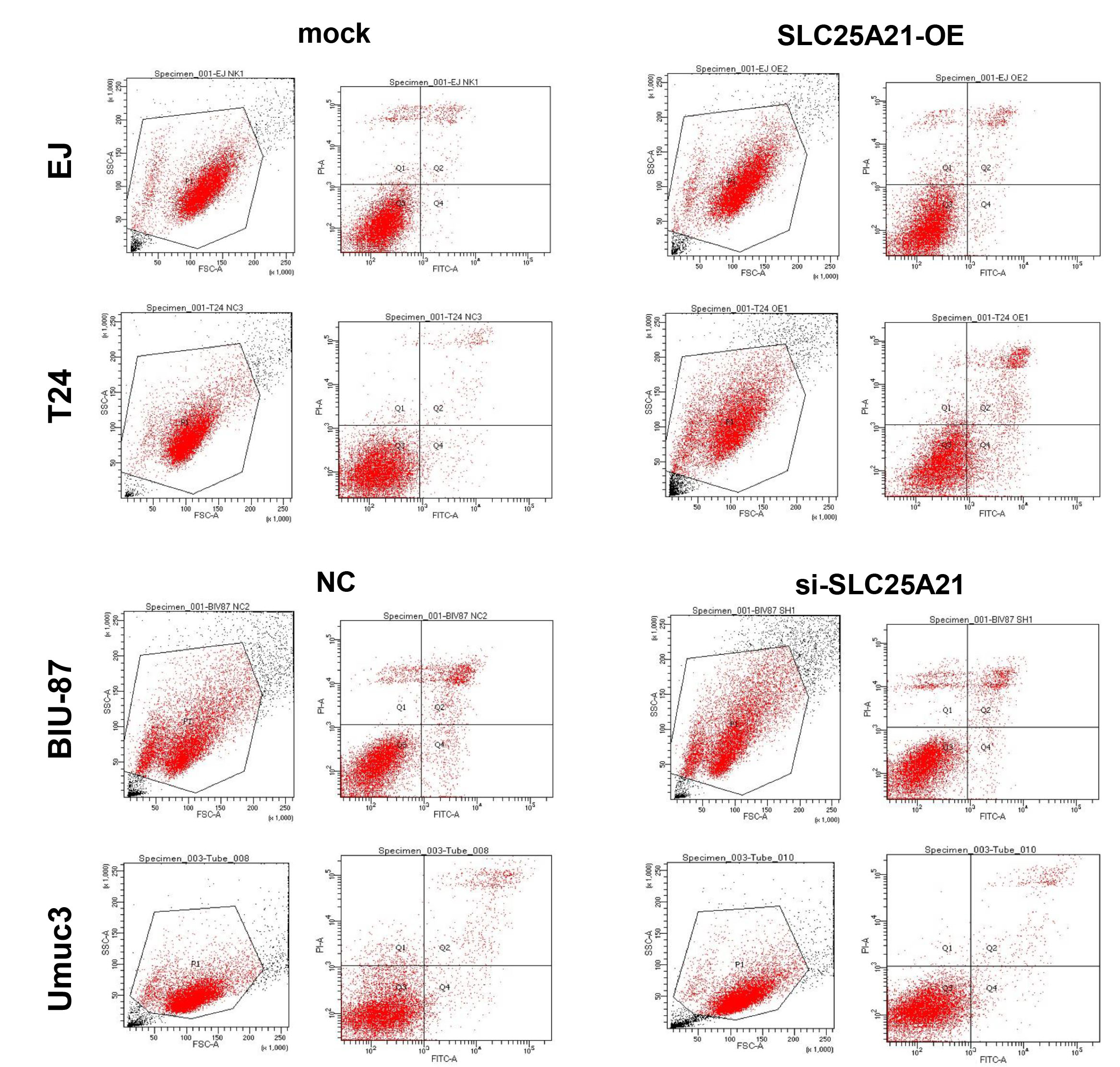


**Figure S3.** **The effect of SLC25A21 on cell apoptosis in BCa cells**
